# Detection of SO_2_F_2_ Using a Photoacoustic Two-Chamber Approach

**DOI:** 10.3390/s24010191

**Published:** 2023-12-28

**Authors:** Hassan Yassine, Christian Weber, Andre Eberhardt, Mahmoud El-Safoury, Jürgen Wöllenstein, Katrin Schmitt

**Affiliations:** 1 Department of Microsystems Engineering IMTEK, University of Freiburg, 79110 Freiburg, Germany; hassan.yassine@imtek.uni-freiburg.de (H.Y.); christian.weber@imtek.uni-freiburg.de (C.W.); juergen.woellenstein@imtek.uni-freiburg.de (J.W.); 2 Fraunhofer Institute for Physical Measurement Techniques IPM, 79110 Freiburg, Germany; andre.eberhardt@ipm.fraunhofer.de (A.E.); mahmoud.el-safoury@ipm.fraunhofer.de (M.E.-S.)

**Keywords:** photoacoustic spectroscopy, sulfuryl difluoride (SO_2_F_2_) detection, two-chamber photoacoustic sensors

## Abstract

The wide use of sulfuryl difluoride (SO_2_F_2_) for termite control in buildings, warehouses and shipping containers requires the implementation of suitable sensors for reliable detection. SO_2_F_2_ is highly toxic to humans and the environment, and moreover, it is a potent greenhouse gas. We developed two photoacoustic two-chamber sensors with the aim to detect two different concentration ranges, 0–1 vol.-% SO_2_F_2_ and 0–100 ppm SO_2_F_2_, so that different applications can be targeted: the sensor for high concentrations for the effective treatment of buildings, containers, etc., and the sensor for low concentrations as personal safety device. Photoacoustic detectors were designed, fabricated, and then filled with either pure SO_2_F_2_ or pure substituent gas, the refrigerant R227ea, to detect SO_2_F_2_. Absorption cells with optical path lengths of 50 mm and 1.6 m were built for both concentration ranges. The sensitivity to SO_2_F_2_ as well as cross-sensitivities to CO_2_ and H_2_O were measured. The results show that concentrations below 1 ppm SO_2_F_2_ can be reliably detected, and possible cross-sensitivities can be effectively compensated.

## 1. Introduction

The extensive use of sulfuryl difluoride (SO_2_F_2_), mostly as replacement for methyl bromide, for termite control in buildings, warehouses and shipping containers has led to a significant increase in global emissions and therefore the atmospheric mixing ratios from 1.8 parts-per-trillion (ppt) in 2011 to 2.6 ppt in 2019 [1,2]. SO_2_F_2_ is highly toxic to humans [3] and the environment, and moreover, it is a potent greenhouse gas with a global warming potential of 4780 and an atmospheric lifetime of 36 years [4,5]. Due to its toxicity, occupational safety requires the monitoring of SO_2_F_2_, e.g., in workplaces or during the fumigation of buildings. The occupational exposure limit (OEL) of SO_2_F_2_ is 5 parts-per-million (ppm) and an immediate danger for life and health starts from 200 ppm [3], so suitable sensors need to be able to monitor the lower ppm range, better reaching the parts-per-billion (ppb) level. A large variety of sensors has been reported in recent years. Many of them are chemical sensors based on different material complexes, e.g., Mo-, W-, Hf, or Co-based [6,7,8,9,10], NiO- or TiO-based [11,12,13], or they implement transition metals [14,15]. Generally, chemical sensors are designed to sense the target gas in low concentration ranges, yet the material systems often suffer from cross-sensitivities to other gases and do not have sufficient long-term stability. Apart from their detection using chemical sensors, gases often can be monitored using optical sensors, making use of their characteristic absorption properties [16,17]. Gas absorption can be observed in the ultraviolet but also in the visible and infrared wavelength range. Simple sensors employ the non-dispersive infrared (NDIR-) principle with broadband sources and detectors and optical filters for measuring the absorption of the target gas within a defined optical path. More complex and cost-intensive, but also up to several decades more sensitive, are laser-based systems. Both principles have already been proposed for SO_2_F_2_ detection [18,19,20,21]. NDIR sensors can be designed in a size- and cost-efficient way but often lack sensitivity, whereas laser-based systems are more complex but show better performance. Laser-based systems are often still laboratory-based due to their complexity, which limits their applicability in field applications.

Therefore, we decided to employ the principle of photoacoustic detection in our SO_2_F_2_ sensor. Photoacoustic devices are highly selective and, depending on their mode of operation, also highly sensitive with detection limits in the ppb range, which makes such devices highly suitable for many application fields [22,23]. Generally, resonant devices can achieve such low detection limits, but they are much more complex and expensive than non-resonant sensors. Resonant photoacoustic sensors have already been proposed for SO_2_F_2_ sensing but are restricted to laboratory use [24,25,26,27,28]. If the modulation of the light source is operated at a frequency that is much smaller than the smallest resonance frequency of the photoacoustic cell (PA) cell, this is referred to as non-resonant operation. In this case, the sound wave has a wavelength much larger than the dimensions of the cell. This means that no standing wave can form in the cell, as is the case for resonant devices [29]. Non-resonant systems, although they only show detection limits in the ppm range, might be more suitable for field use. Such sensors can be equipped with micro-electromechanical system (MEMS) components, and they are already available commercially, e.g., for CO_2_ sensing from the companies Infineon or Sensirion.

**Figure 1:**
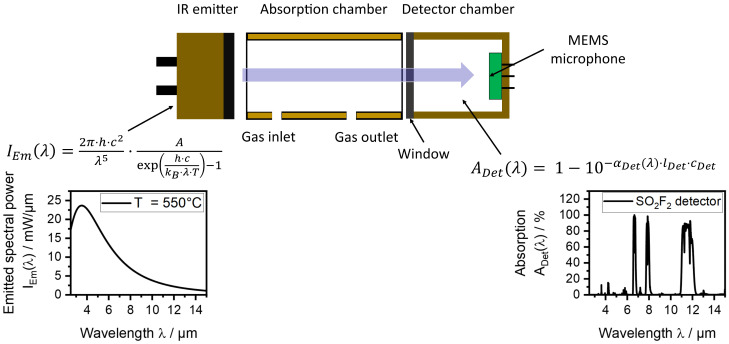
Schematic illustration of the photoacoustic sensor setup using the two-chamber approach. The graphs show the emitted spectral power I_Em_(λ) of the JSIR 350-4-AL-R-D6.0-2-A7 IR emitter used (Micro-Hybrid Electronic GmbH, Germany) between 2.5 µm and 15 µm at an emitter temperature of 550 °C (electrical input power of 0.36 W), calculated using Planck’s law from [31] and the information provided in the datasheet [32]. Radiation losses resulting from the transmission through the window of the IR emitter were not considered. It shows the absorption A_Det_(λ) of a SO_2_F_2_ detector with a detector length l_Det_ of 1.5 mm, calculated using the Beer–Lambert law.

We propose to use a non-resonant, two-chamber photoacoustic setup as schematically illustrated in Figure 1. In this setup, we employ an IR emitter, a measurement/absorption chamber to be filled with the gas mixture to be measured and a second chamber (detection chamber) filled with the target gas or a suitable substitute gas. In our previous publication, we have reported detailed simulations on the sensor design and optical path length, considering different gases for the detector chamber [30]. The idea is to use a non-toxic substituent gas with similar absorption characteristics in the photoacoustic detector chamber for indirect, but still selective, SO_2_F_2_ sensing. Photoacoustic detectors were designed, fabricated and then filled with either pure SO_2_F_2_ or pure substituent gas, the refrigerant 1,1,1,2,3,3,3-heptafluoropropane (R227ea), to detect SO_2_F_2_. Absorption cells with optical path lengths of 50 mm and 1.6 m were built for both concentration ranges. The sensitivity to SO_2_F_2_ as well as cross-sensitivities to carbon dioxide (CO_2_) and water vapor (H_2_O) were measured. We also compare the results to our simulations and other SO_2_F_2_ photoacoustic sensors reported in the literature. The results showed that our photoacoustic sensor with the absorption cell with an optical path length of 1.6 m can monitor the OEL of SO_2_F_2_ and can therefore be deployed in various applications in the future.

## 2. Materials and Methods

### 2.1. Photoacoustic Sensors Setups

Our photoacoustic two-chamber sensors consist of an infrared radiation source, an absorption cell and a photoacoustic detector. The JSIR 350-4-AL-R-D6.0-2-A7 IR emitter (Micro-Hybrid Electronic GmbH, Germany) offers a sufficiently high optical output power and spectral range for the intended SO_2_F_2_ sensor. Figure 1 shows the working principle of our sensor and its components schematically. The graphs illustrate the emitted spectral power of the IR emitter I_Em_(λ) between 2.5 µm and 15 µm at an emitter temperature of 550 °C (electrical input power of 0.36 W) calculated using Planck’s law from [31] and the information provided in the datasheet [32]. Radiation losses resulting from the transmission through the window of the IR emitter were not considered. Furthermore, the absorption of an SO_2_F_2_ detector is shown, calculated according to the Beer–Lambert law from [33] with a detector length of 1.5 mm. The transmission through the detector window was considered here. In the absence of SO_2_F_2_ in the absorption chamber, the detector signal is maximum, and it decreases when SO_2_F_2_ is present. Due to the absorption of part of the IR radiation by the SO_2_F_2_ molecules in the absorption chamber, the absorption by SO_2_F_2_ in the detector decreases and thus also the detector signal decreases.

**Figure 2:**
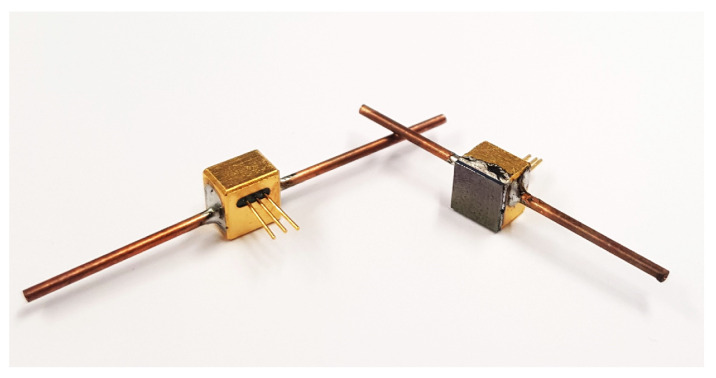
Picture showing the back side (**left**) and front side with detector window (**right**) of the constructed detector chamber (5 mm × 5 mm × 4 mm). Two copper tubes (1 mm × 0.5 mm) were soldered into the sides of the detector chamber to fill the detector with gas and a 500 µm double side polished <110> 4-inch Si window with a metallization layer was soldered to the detector. The MEMS microphone is integrated into the detector chamber, and the contacts are led outside the detector chamber via a gas-tight glass feedthrough.

In addition to the choice of the radiation source, both the geometry of the photoacoustic detector and that of the absorption cell play an important role for the sensor signal, the sensitivity of the sensor and the resulting detection limit. The detector (5 mm × 5 mm × 4 mm) shown in Figure 2 was milled from 1.1730 steel and then nickel-plated (d = 5 µm) and gold-plated (d = 1.5 µm) by SIMEK Silikat-, Metall- und Kunstofftechnik GmbH, Vilsbiburg, Germany. A SPV1840LR5H-B MEMS microphone (Knowles Electronics, Santa Clara, CA, USA) was integrated into the detector chamber, and its contacts were led outside the detector chamber via a gas-tight glass feedthrough manufactured also by SIMEK GmbH. Afterwards, copper tubes (1 mm × 0.5 mm) were soldered into the sides of the detector chamber to fill the detector with gas. A 500 µm double side polished <110> 4-inch Si wafer was used as a detector window. A metallization layer for soldering was applied onto the wafer, and the wafer was then cut into quadratic windows (4.8 mm × 4.8 mm). The metallized Si window was soldered to the detector, sealing the gas cavity.

The simulation results in [30] showed that the refrigerant R227ea could be a suitable substituent gas for SO_2_F_2_ in the detector gas filling. Thus, the detectors were prepared and then flushed with either 100 vol.-% SO_2_F_2_ or 100 vol.-% R227ea. After that, the ends of the filling tubes of the detectors were crimped and then soldered.

**Figure 3:**
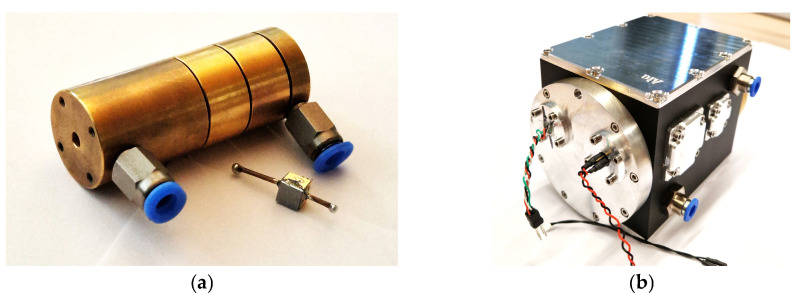
(**a**) Picture of the absorption cell (20 mm × 50 mm) made of brass, consisting of four segments together with a photoacoustic detector in the foreground. The optical path length is 50 mm and the diameter of the optical path is 3 mm. The photoacoustic detector was mounted to the first end of the absorption cell and the IR emitter to the second end. (**b**) Overall setup of the multi-pass White cell with an optical path length of 1.6 m, with the IR emitter and the photoacoustic detector. The IR emitter was mounted in the opening on the right side, while the detector was mounted in the opening on the left side.

For the concentration range 0–1 vol.-% SO_2_F_2_, an absorption cell (Figure 3a) with an optical path length of 50 mm and an internal diameter of 3 mm was designed. It was manufactured from brass and subsequently chemically Au-coated. For manufacturing reasons, the absorption cell consists of four individual segments. The photoacoustic detector was then fixed to the first end of the absorption cell and the IR emitter to the other end. For the concentration range 0–100 ppm SO_2_F_2_, a multi-pass White cell with an optical path length of 1.6 m was designed according to [34] and built. As the sensor signal of a two-chamber photoacoustic sensor changes according to the Beer–Lambert law, extending the optical path length *l* in Equation (1) increases the sensitivity. Hence, lower concentrations *c* can be detected [33].

(1) $$I=I_{0}\cdot 10^{-\alpha \cdot l\cdot c},$$

where *I* and *I*_0_ are the radiation intensity and initial radiation intensity, respectively, and *α* is the decadic absorption coefficient. Figure 3b shows a photograph of the overall setup of the multi-pass cell with the IR emitter and the photoacoustic detector. The IR emitter was mounted in the opening on the right side while the detector was mounted in the opening on the left side.

### 2.2. SO_2_F_2_ Gas Measurements

All measurements were performed with an electronic board similar to the one described in [35]. The analog and digital signal processing, including a digital lock-in algorithm, as well as the control and modulation of the emitter were performed with this electronic board. The effect of the modulation frequency of both photoacoustic detectors, the SO_2_F_2_ detector and the R227ea detector, on the sensor signal was investigated. Both measurements were performed in a nitrogen (N_2_) atmosphere using the sensors with the 50 mm absorption cell.

After that, measurements determining the sensitivity of both photoacoustic detectors to SO_2_F_2_ were performed. First, SO_2_F_2_ concentrations between 0 and 1000 ppm in steps of 100 ppm and a duration of 30 min for each SO_2_F_2_ step were applied (cf. Figure S1). After that, SO_2_F_2_ concentrations below 400 ppm, in steps of 50 ppm also lasting 30 min each, were applied. The IR emitter was modulated with a frequency of 35 Hz in both systems and the lock-in time constant was set to 20 s. In addition, measurements of possible cross-sensitivities of both photoacoustic detectors to CO_2_ and H_2_O were performed. The same driver current, modulation frequency and lock-in time constant as in the previous measurement were used. CO_2_ concentrations of 400 ppm, 600 ppm, 800 ppm and 1000 ppm as well as relative humidity contents of 20% r.H. to 80% r.H. (at T = 30 °C) in 20% r.H. steps were applied in these measurements.

Based on the observed sensor performance, experiments with the multi-pass White cell (l = 1.6 m) were set up. SO_2_F_2_ concentrations between 0 and 100 ppm in concentration steps of 10 ppm and a duration of 1h each were applied in this measurement (cf. Figure S2). Furthermore, SO_2_F_2_ concentrations of 2 ppm, 10 ppm, 15 ppm and 20 ppm with the same duration were applied additionally. The IR emitter was modulated with a frequency of 35 Hz and the time constant of the lock-in filter was set to 20 s. Measurements of cross-sensitivities of both photoacoustic detectors to CO_2_ and H_2_O were also carried out. CO_2_ concentrations of 400 ppm, 600 ppm, 800 ppm and 1000 ppm as well as relative humidity contents of 10% r.H. to 60% r.H. (at T = 30 °C) in 10% r.H. steps were used as concentration setpoints.

## 3. Results

### 3.1. Sensor Setup for the Measuring Range 0–1 vol.-% SO_2_F_2_

#### 3.1.1. Characterization of the Sensitivity of the Sensors to SO_2_F_2_

**Figure 4:**
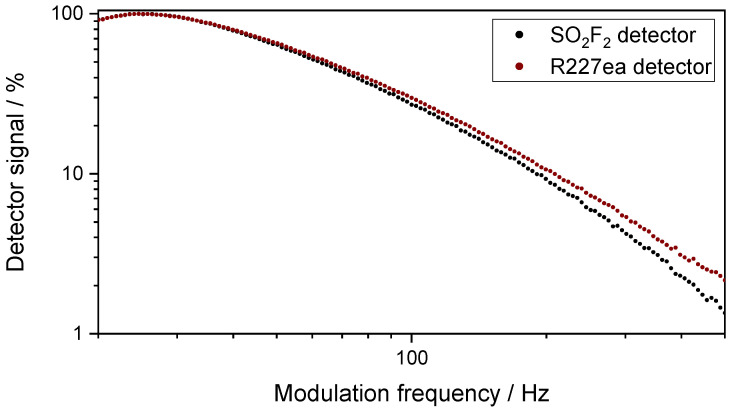
Variation in the signal of the SO_2_F_2_ photoacoustic detector and the R227ea photoacoustic detector with respect to the change in the modulation frequency of the IR emitter in the range between 20 Hz and 500 Hz. The detector signal decreases with the increase in modulation frequency. These measurements were performed with the sensor setup with the 50 mm absorption cell in N_2_ atmosphere.

The dependence of the signal of both photoacoustic detectors (SO_2_F_2_ detector and R227ea detector) on the modulation frequency of the IR emitter is shown in Figure 4. Both photoacoustic detectors show a similar change with modulation frequency. Between 20 Hz and 35 Hz, the signal of both detectors is higher than 85% of its maximum value. At 500 Hz, it decreases to less than 5%. This was expected due to the decrease in the modulation depth of the IR emitter with higher frequencies as well as the 1/f signal decrease of photoacoustic detectors. At the same time, the 1/f noise of the microphone decreases with the modulation frequency. So, as a trade-off between the absolute signal of the photoacoustic detectors and their noise, a modulation frequency of 35 Hz was used in all measurements.

**Figure 5:**
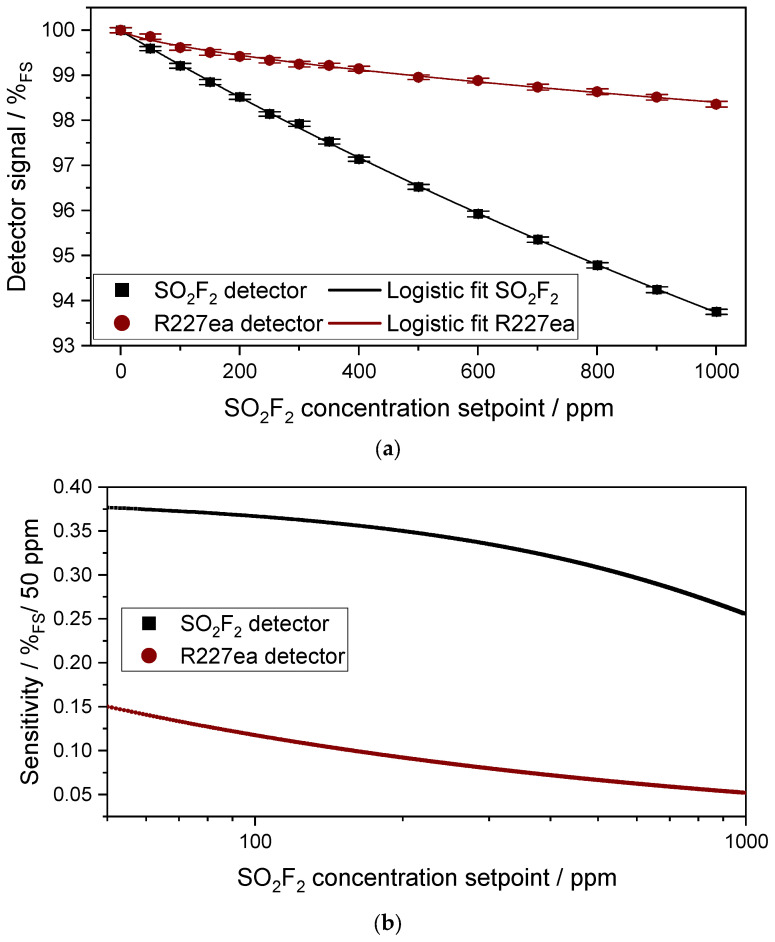
(**a**) Variation in the sensor signal of both photoacoustic detectors as a function of the SO_2_F_2_ concentration change in the absorption cell (l = 50 mm). A logistic function as in Equation (2) describes the detector response well. (**b**) Sensitivity of both photoacoustic detectors in %_FS_/50 ppm in concentration range between 50 and 1000 ppm SO_2_F_2_.

Figure 5a shows the signal of the SO_2_F_2_ photoacoustic detector as well as that of the R227ea photoacoustic detector with respect to the concentration change of SO_2_F_2_ in the 50 mm absorption cell between 0 and 1000 ppm. The higher the given concentration, the lower the detector signal is. Both photoacoustic detectors show a non-linear response to the concentration change in SO_2_F_2_ in the absorption cell. A logistic function as in Equation (2) describes the detector response well:

(2) $$y=\left(\frac{a-b}{1+{\left(\frac{X}{x_{0}}\right)}^{p}}\right)+b,$$

where, *a*, *b*, *x*_0_ and *p* are fit parameters. The relative signal change of the SO_2_F_2_ photoacoustic detector to the SO_2_F_2_ concentration change in the absorption cell is higher than the relative signal change of the R227ea photoacoustic detector to the SO_2_F_2_ concentration change in the absorption cell. In the presence of 1000 ppm SO_2_F_2_ in the absorption cell, the SO_2_F_2_ photoacoustic detector shows a detector signal of 93.75%_FS_ (FS = full scale), whereas that of R227ea decreases to 98.35%_FS_. The detector signal of the SO_2_F_2_ detector was 99.59%_FS_ at 50 ppm SO_2_F_2_, while that of R227ea was 99.85%_FS_. The 3σ noise was 0.16%_FS_ for the SO_2_F_2_ detector at 0 ppm SO_2_F_2_, while that of the R227ea was 0.174%_FS_. Considering the 3σ noise of both detectors and the sensitivity of the detectors to different SO_2_F_2_ concentrations, the detection limit and the 3σ resolution of the sensors can be determined.

**Figure 6:**
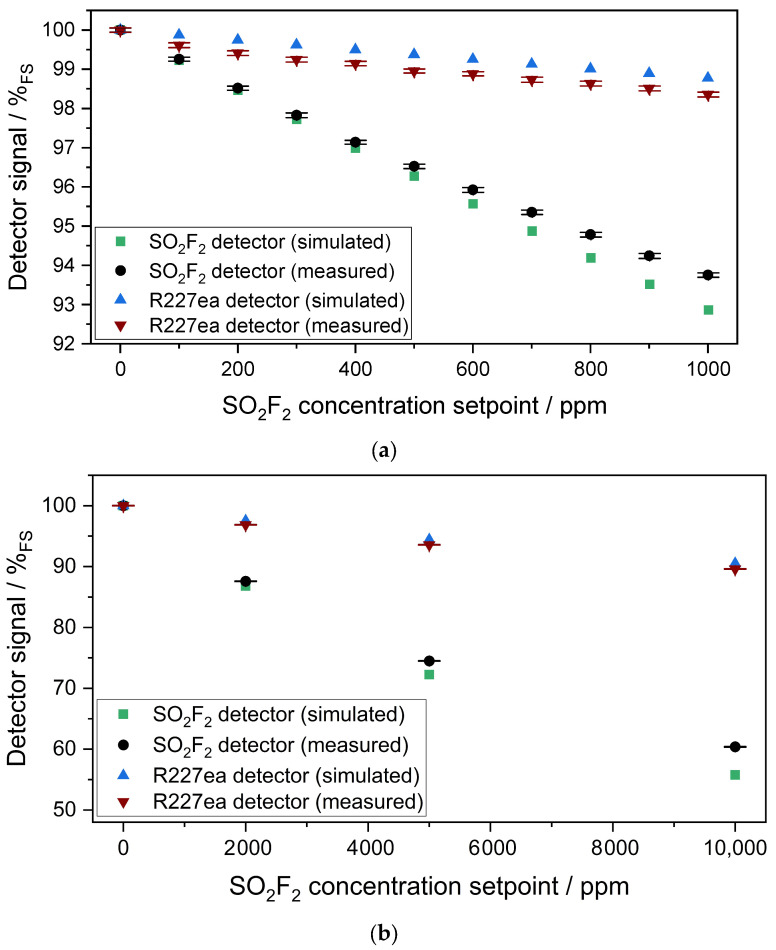
(**a**) Variation in the measured and the simulated signals of both photoacoustic detectors as a function of the SO_2_F_2_ concentration in the absorption cell (l = 50 mm) between 0 and 1000 ppm as well as that at SO_2_F_2_ concentrations of 2000 ppm, 5000 ppm and 10,000 ppm in the absorption cell (l = 50 mm) in (**b**).

Figure 5b shows the sensitivity of both photoacoustic detectors in %_FS_/50 ppm to SO_2_F_2_ in the concentration range of 50–1000 ppm SO_2_F_2_. The first derivative of the logistic fit with respect to the concentration yields the sensitivity of the photoacoustic detectors versus the concentration. The derivative of the fit is chosen over the derivative raw data for obtaining continuous data with less discretization at the cost of some accuracy. The sensitivity of the SO_2_F_2_ photoacoustic sensor in the range of 0–1000 ppm SO_2_F_2_ is 2.5× to 6× higher than the sensitivity of the R227ea detector. The higher the SO_2_F_2_ setpoint concentration, the lower the sensitivity of both photoacoustic detectors. Considering the 3σ noise of both detectors as well as the sensitivity of both detectors to SO_2_F_2_ concentrations shown in Figure 5b, detection limits of 20 ppm for the SO_2_F_2_ detector and 60 ppm for the R227ea detector at 0 ppm SO_2_F_2_ were determined. The 3σ resolution for the SO_2_F_2_ detector is 34 ppm at 1000 ppm SO_2_F_2_, while it is 180 ppm for the R227ea detector. This is due to the lower sensitivities at higher SO_2_F_2_ concentrations. The response time t_90_ of the sensor is 20 s, which is dominated here by the lock-in integration time required for the measurements and not by the volume of the absorption chamber. If the volume of the absorption chamber (approx. 360 µL) dominated here, the sensor would have to have a time constant t_90_ of less than 0.5 s at an SO_2_F_2_ flow rate of 0.5 L/min. Figure 6a,b compare the simulated and the measured signal of the two photoacoustic detectors as a function of the SO_2_F_2_ concentration in the range of 0–10,000 ppm. An in-depth explanation on how the simulations were conducted can be found in [30]. The simulations consider the Beer–Lambert law seen in Equation (1) spectrally to determine the absorption in the detector chamber and at the absorption chamber. There is a difference between the simulated and measured signal of both photoacoustic detectors as can be noticed from Figure 6a,b. The higher the SO_2_F_2_ concentration setpoint in the absorption cell, the greater the difference between simulated and measured signal. The difference in the simulated and the measured signal of the SO_2_F_2_ photoacoustic detector is probably due to the fact that the simulations were calculated under ideal conditions by assuming that the detector chamber was filled with 100 vol.-% SO_2_F_2_. In reality, the detector chamber might still contain traces of interfering gases generated during the soldering, so that it is not filled with pure SO_2_F_2_. This leads to a change in the sensitivity of the SO_2_F_2_ photoacoustic detector to various SO_2_F_2_ concentrations in the absorption cell. The utilized IR emitter has a Si window with an anti-reflective coating which was also not considered in the simulation. Furthermore, flux residues or dirt particles on the surface of the Si window affect the IR transmission through the window, which leads to a change in the sensitivity of the detector. In addition, the absorption cell consists of four segments, which significantly increases the probability of IR radiation loss. These could also be reasons why the R227ea photoacoustic detector has a higher sensitivity in the measurements than in the simulations. Moreover, the IR radiation rays could pass the absorption cell diagonally via several reflections, causing an extension of the effective optical path length and thus leading to a higher sensitivity.

#### 3.1.2. Characterization of the Cross Sensitivity of the Sensors to CO_2_ and H_2_O

**Figure 7:**
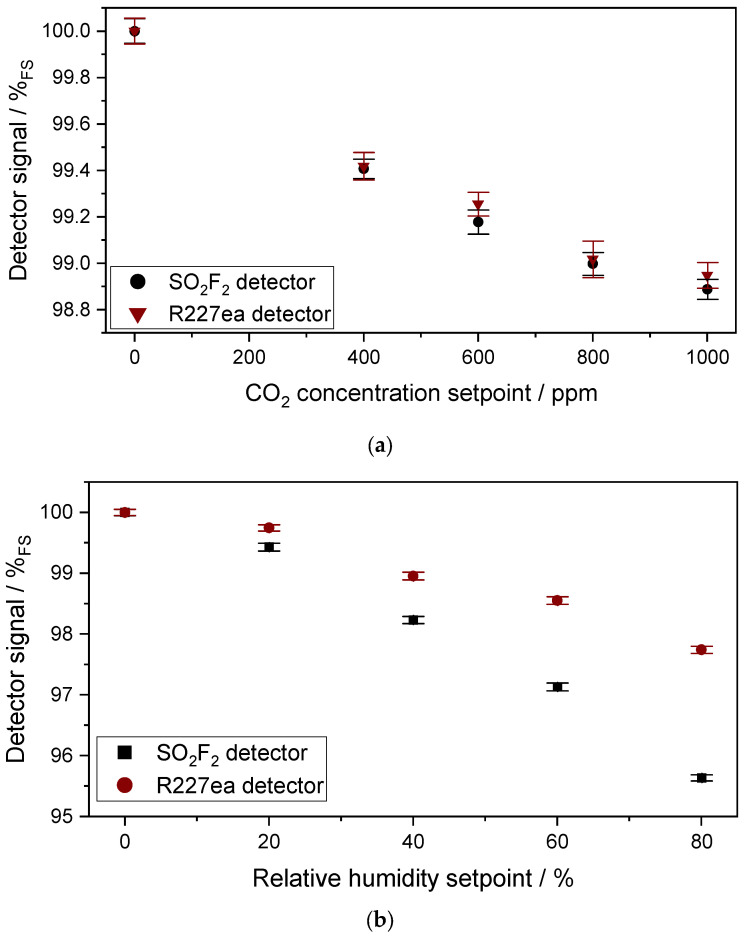
(**a**) Measured signal of both photoacoustic detectors with 400 ppm, 600 ppm, 800 ppm and 1000 ppm CO_2_ in the absorption cell (l = 50 mm) and (**b**) 20%, 40%, 60% and 80% relative humidity (at T = 30 °C).

The results of the measurements regarding the cross-sensitivity of the two photoacoustic detectors towards CO_2_ and H_2_O are shown in Figure 7a,b. The SO_2_F_2_ detector shows slightly higher cross-sensitivity to CO_2_ than the R227ea detector. This is due to SO_2_F_2_ having more overlapping absorption lines with CO_2_ than R227ea. In addition, interfering gases in the detectors may increase the cross-sensitivities to other gases as well. The relative signal change of the SO_2_F_2_ photoacoustic detector when the CO_2_ concentration in the absorption cell was changed from 400 ppm to 1000 ppm is about 0.5%_FS_, which corresponds to a similar relative signal change when the SO_2_F_2_ concentration in the absorption cell was changed from 0 to 65 ppm. In contrast, when the CO_2_ concentration in the absorption cell changed from 400 ppm to 1000 ppm, the relative signal change of the R227ea photoacoustic detector was about 0.4%_FS_, corresponding to a similar relative signal change when the SO_2_F_2_ concentration in the absorption cell was changed from 0 to 150 ppm. Depending on the application field of the sensor, an IR filter that blocks the wavelength range below 5 µm could be mounted onto the IR emitter to minimize this cross-sensitivity.

A change in relative humidity has a higher effect on the signal than CO_2_ in the sensors with both detectors. The sensor with the SO_2_F_2_ detector shows higher cross-sensitivity to humidity than the R227ea photoacoustic detector. The relative signal change of the SO_2_F_2_ photoacoustic detector when the relative humidity in the absorption cell was changed from 40% r.H. to 80% r.H. is about 2.6%_FS_, which corresponds to an equivalent of 360 ppm SO_2_F_2_. The relative signal change of the R227ea detector following the relative humidity change from 40% r.H. to 80% r.H. is about 1.2%_FS_, which corresponds to a SO_2_F_2_ concentration change from 0 to 650 ppm. One strategy to mitigate this cross-sensitivity would be the implementation of a humidity sensor into the system for compensation.

### 3.2. Sensor for the Measuring Range 0–100 ppm SO_2_F_2_

#### 3.2.1. Characterization of the Sensitivity of the Sensors to SO_2_F_2_

**Figure 8:**
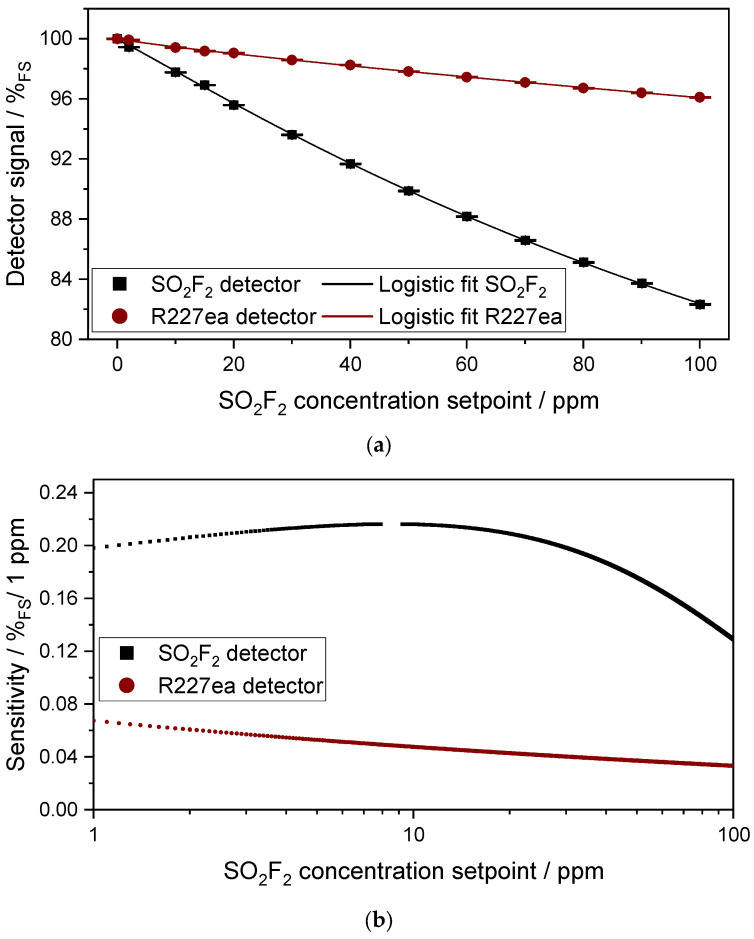
(**a**) Variation in the sensor signal of both photoacoustic detectors as a function of the SO_2_F_2_ concentration change in the absorption cell (l = 1.6 m). A logistic function as in Equation (2) describes the detector response well. (**b**) Sensitivity of both photoacoustic detectors in %_FS_/1ppm in concentration range between 1 and 100 ppm SO_2_F_2_.

As with the sensor setup with the 50 mm absorption cell, which was analyzed in detail in the previous subsection, experiments were also carried out with the sensor setup with the 1.6 m multi-pass absorption cell. Figure 8a displays the signal of both photoacoustic detectors with respect to the concentration change of SO_2_F_2_ between 0 and 100 ppm in the 1.6 m multi-pass absorption cell. As in the experiments with the 50 mm cell, both photoacoustic detectors again show a non-linear response to the concentration change in SO_2_F_2_. In the presence of 100 ppm SO_2_F_2_ in the absorption cell, the SO_2_F_2_ photoacoustic detector shows a detector signal of 82.34%_FS_, whereas that of R227ea decreases to 96.1%_FS_. Considering the sensitivity results of the two photoacoustic detectors displayed in Figure 8b, as well as the 3σ noise of 0.08%_FS_ of the SO_2_F_2_ detector and 0.11%_FS_ of the R227ea detector, a detection limit of 0.5 ppm can be achieved with the SO_2_F_2_ detector and 2 ppm with the R227ea detector. The 3σ resolution of the SO_2_F_2_ detector is 1.25 ppm at 100 ppm SO_2_F_2_, while it is 4.25 ppm for the R227ea detector. This is due to the lower sensitivities at higher SO_2_F_2_ concentrations. The response time t_90_ of the sensor is 40 s at a SO_2_F_2_ flow rate of 2 L/min, which is dominated here by the volume of the absorption chamber (approx. 500 mL) and the time required for the gas exchange in the chamber to reach the SO_2_F_2_ concentration setpoint.

**Figure 9:**
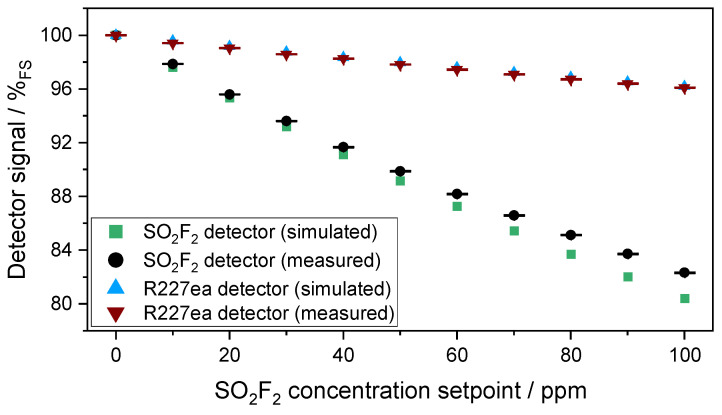
Variation in the measured and the simulated signals of both photoacoustic detectors as a function of the SO_2_F_2_ concentration change in the absorption cell (l = 1.6 m) between 0 and 100 ppm.

Figure 9 compares the simulated and measured signal of the two photoacoustic detectors. As in the previous experiments with the 50 mm absorption cell, there is a difference between the simulated and measured signal of both photoacoustic detectors. However, the difference between the simulated and measured signal of both photoacoustic detectors seems to be smaller here with the 1.6 m cell than with the 50 mm cell. This seems feasible because the IR radiation losses at characteristic wavelengths are larger in the 50 mm absorption cell than in the 1.6 m multi-pass absorption cell, which in turn has an influence on the signal change. Due to manufacturing reasons, the 50 mm absorption cell consists of four segments, which leads to higher IR radiation losses. In addition, the surface quality of the mirrors of the multi-pass absorption cell is much better than the surface quality of the optical path of the 50 mm absorption cell, which leads to lower radiation losses. Of course, the reasons already mentioned in the discussion with the 50 mm absorption cell play a role here as well.

#### 3.2.2. Characterization of the Cross-Sensitivity of the Sensors to CO_2_ and H_2_O

**Figure 10:**
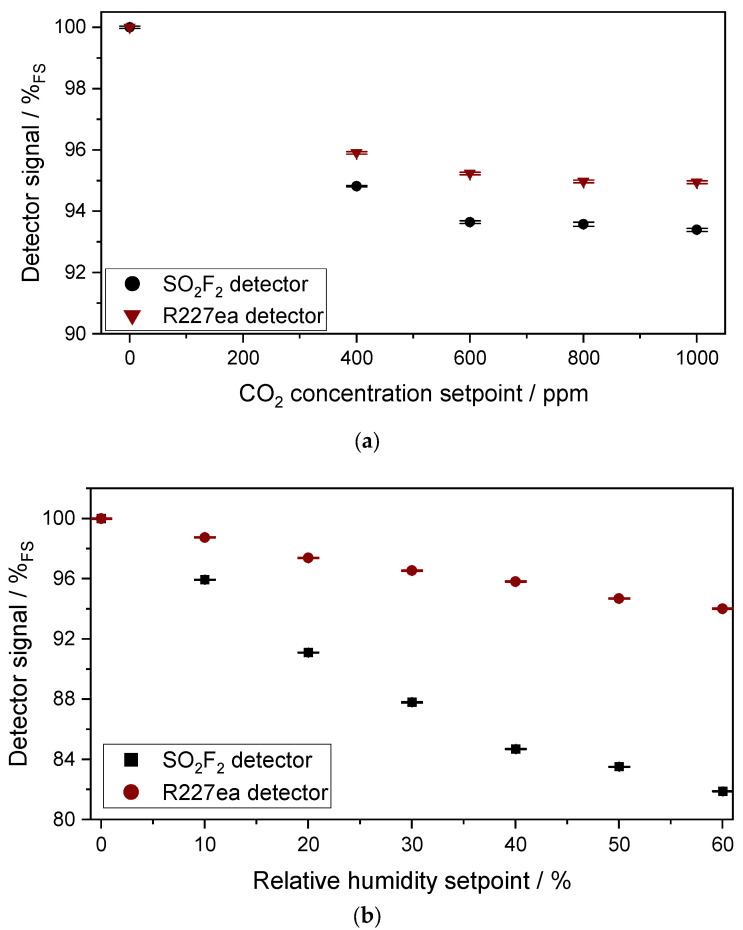
(**a**) Measured signal of both photoacoustic detectors to 400 ppm, 600 ppm, 800 ppm and 1000 ppm CO_2_ in the absorption cell (l = 1.6 m) and (**b**) to 10%, 20%, 30%, 40%, 50% and 60% relative humidity (at T = 30 °C).

Figure 10a,b show the results of the measurements on the cross-sensitivity of the two detectors to CO_2_ and H_2_O, with the optical path length of 1.6 m. The effect of the cross-sensitivity becomes higher compared to the 50 mm cell. The relative signal change of the SO_2_F_2_ photoacoustic detector when the CO_2_ concentration was changed from 400 ppm to 1000 ppm in the absorption cell is about 1.4%_FS_, which corresponds to a concentration change of SO_2_F_2_ from 0 to 6.3 ppm in the absorption cell. In contrast, when the CO_2_ concentration changed from 400 ppm to 1000 ppm in the absorption cell, the relative signal change of the R227ea photoacoustic detector was about 0.9%_FS_, corresponding to a concentration change of SO_2_F_2_ from 0 to 19.9 ppm.

As before with the 50mm cell, the change in relative humidity has a much higher effect on the signal of both photoacoustic detectors than CO_2_. The SO_2_F_2_ photoacoustic detector shows higher cross-sensitivity to humidity than the R227ea photoacoustic detector. The relative signal change of the SO_2_F_2_ photoacoustic detector with a humidity change from 30% to 60% is about 5.9%_FS_, which corresponds to a concentration change of SO_2_F_2_ from 0 to 27.4 ppm. The signal change of the sensor with the R227ea detector with a relative humidity change from 30% to 60% is about 2.5%_FS_, which corresponds to a concentration change of SO_2_F_2_ from 0 to 59 ppm. The response of the SO_2_F_2_ detector to H_2_O is higher than the R227ea detectors, but due to the higher sensitivity of the SO_2_F_2_ detector to SO_2_F_2_ than R227ea, the equivalent cross-sensitivity of the SO_2_F_2_ detector is smaller.

## 4. Conclusions

Two photoacoustic sensor setups for the detection of SO_2_F_2_ in two concentration ranges (0–1 vol.-% SO_2_F_2_ and 0–100 ppm SO_2_F_2_) were presented. Both sensors are based on the photoacoustic two-chamber approach, consisting of an IR emitter, an absorption cell and a photoacoustic detector. The first sensor setup has an absorption cell with an optical length of 50 mm for detecting SO_2_F_2_ in the concentration range of 0–1 vol.-% SO_2_F_2_, while the second setup has a multi-pass absorption cell with an optical path length of 1.6 m for detecting SO_2_F_2_ in the concentration range of 0–100 ppm. The photoacoustic detector was designed as a hermetically tight gas cavity that was equipped with a MEMS microphone and sealed by soldering a Si window. After that, the detectors were filled with either pure SO_2_F_2_ or a substituent gas, the refrigerant R227ea. The sensitivity to SO_2_F_2_ and cross-sensitivity to CO_2_ and H_2_O were measured for both photoacoustic detectors with both absorption cells and strategies for their mitigation discussed. Using the sensor setup with the 50 mm absorption cell, a detection limit of 20 ppm was obtained with the SO_2_F_2_ photoacoustic detector and 60 ppm with the R227ea photoacoustic detector. With the multi-pass absorption cell, a detection limit of 0.5 ppm was obtained with the SO_2_F_2_ photoacoustic detector and 2 ppm with the R227ea photoacoustic detector. Although these detection limits are approximately two decades higher than those that can be achieved with resonant photoacoustic systems [25,29], our sensor is able to monitor the OEL in different applications in the future. A direct comparison with sensing devices relying on chemical principles (cf. [6,7,8,9,10,11,12,13,14,15]) is difficult because these studies investigated the sensing ability of different materials but did not yet set up sensor devices. The next steps in our sensor development include improvements in sensor integration and the testing of the sensor systems in field applications, e.g., in freight containers.

## Data Availability

The data presented in this study are available in this article.

## Supplementary Materials

The following supporting information can be downloaded at https://www.mdpi.com/article/10.3390/s24010191/s1, Figure S1: Sensor response to SO_2_F_2_ using the 50 mm cell; Figure S2: Sensor response to SO_2_F_2_ using the 1.6 m cell.
