# Supplementary material for: Detection of SO2F2 Using a Photoacoustic Two-Chamber Approach
Source: Sensors (Basel). 2023 Dec 28;24(1):191. doi: 10.3390/s24010191 (PMC10781292; doi:10.3390/s24010191)
Supplement: Supplementary file 1 [file sensors-24-00191-s001.zip › sensors-2775783-supplementary.pdf]

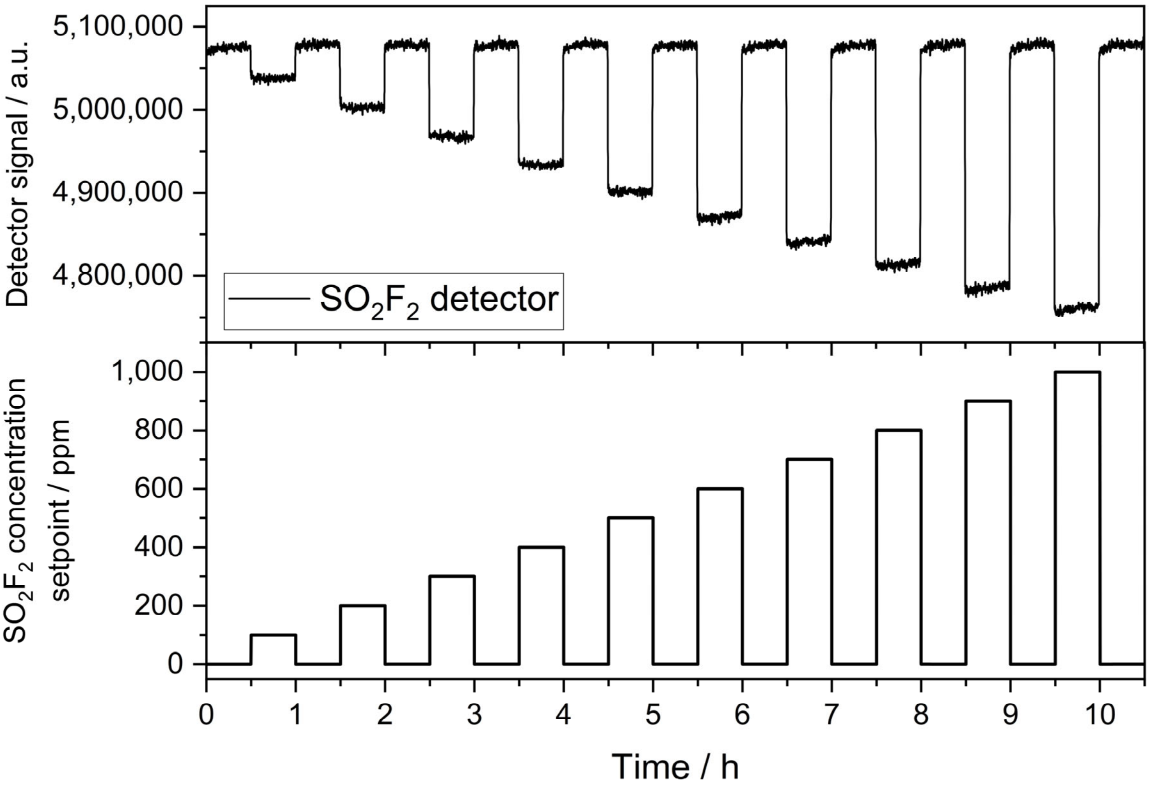

Figure S1. Sensor response to SO_2F_2 using the 50 mm cell.

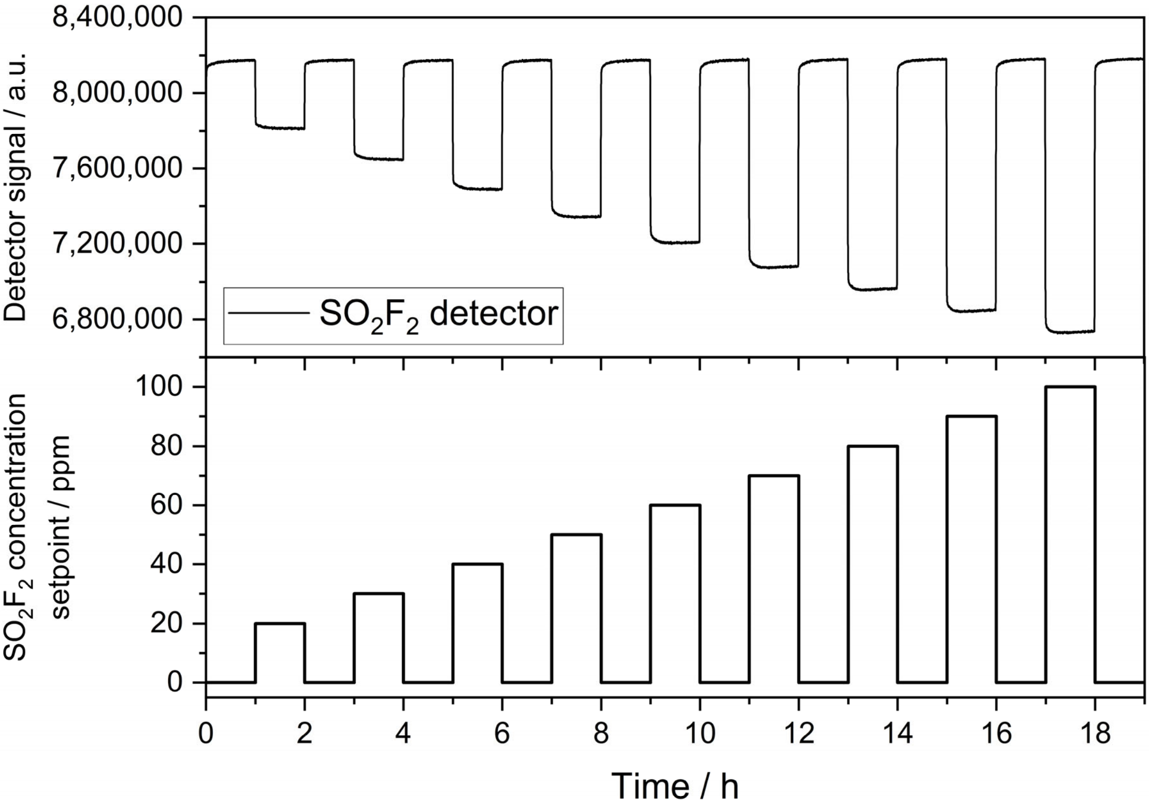

Figure S2. Sensor response to SO_2F_2 using the 1.6 m cell.
